# Stereotactic frame-based biopsy of infratentorial lesions via the suboccipital-transcerebellar approach with the Zamorano-Duchovny stereotactic system—a retrospective analysis of 79 consecutive cases

**DOI:** 10.1007/s00701-024-06036-8

**Published:** 2024-03-23

**Authors:** Manuel Kaes, Christopher Beynon, Karl Kiening, Jan-Oliver Neumann, Martin Jakobs

**Affiliations:** 1 Department of Neurosurgery, Heidelberg University Hospital, Im Neuenheimer Feld 400, 69120 Heidelberg, Germany; 2 Department of Neurosurgery, Division for Stereotactic Neurosurgery, Heidelberg University Hospital, Im Neuenheimer Feld 400, 69120 Heidelberg, Germany; 3 Medical Faculty, Heidelberg University, Grabengasse 1, 69117 Heidelberg, Germany

**Keywords:** Stereotactic biopsy, Posterior fossa, Brainstem, Suboccipital-transcerebellar approach, Cerebellum

## Abstract

**Objective:**

Lesions of the posterior fossa (brainstem and cerebellum) are challenging in diagnosis and treatment due to the fact that they are often located eloquently and total resection is rarely possible. Therefore, frame-based stereotactic biopsies are commonly used to asservate tissue for neuropathological diagnosis and further treatment determination. The aim of our study was to assess the safety and diagnostic success rate of frame-based stereotactic biopsies for lesions in the posterior fossa via the suboccipital-transcerebellar approach.

**Methods:**

We performed a retrospective database analysis of all frame-based stereotactic biopsy cases at our institution since 2007. The aim was to identify all surgical cases for infratentorial lesion biopsies via the suboccipital-transcerebellar approach. We collected clinical data regarding outcomes, complications, diagnostic success, radiological appearances, and stereotactic trajectories.

**Results:**

A total of *n* = 79 cases of stereotactic biopsies for posterior fossa lesions via the suboccipital-transcerebellar approach (41 female and 38 male) utilizing the Zamorano-Duchovny stereotactic system were identified. The mean age at the time of surgery was 42.5 years (± 23.3; range, 1–87 years). All patients were operated with intraoperative stereotactic imaging (*n* = 62 MRI, *n* = 17 CT). The absolute diagnostic success rate was 87.3%. The most common diagnoses were glioma, lymphoma, and inflammatory disease. The overall complication rate was 8.7% (seven cases). All patients with complications showed new neurological deficits; of those, three were permanent. Hemorrhage was detected in five of the cases having complications. The 30-day mortality rate was 7.6%, and 1-year survival rate was 70%.

**Conclusions:**

Our data suggests that frame-based stereotactic biopsies with the Zamorano-Duchovny stereotactic system via the suboccipital-transcerebellar approach are safe and reliable for infratentorial lesions bearing a high diagnostic yield and an acceptable complication rate. Further research should focus on the planning of safe trajectories and a careful case selection with the goal of minimizing complications and maximizing diagnostic success.

## Introduction

Frame-based stereotactic biopsies are the clinical gold standard for acquiring tissue from brain areas for histopathological diagnostics. This technique is often used for lesions with unknown dignity or if located in eloquent or deep-seated areas, even if the lesion itself is considered to be unresectable. Frame-based stereotactic biopsies are known to be safe and reliable. Due to rapid improvements in molecular diagnostic procedures and their increasing significance to treatment decisions, the importance of tissue sampling is growing [5, 11, 13].

Even though radiological methods have evolved significantly, various publications found a high discrepancy comparing the suspected radiological diagnosis with the final histopathological diagnosis after tissue analysis [12, 15, 24]. Furthermore, clear discrimination between the three most common diagnoses of posterior fossa lesions (brain tumor, lymphoma, inflammatory process) is not possible with radiologic diagnostic tools alone [17]. Especially in pediatric patients, the gap between radiological and histopathological diagnosis is well-described [7, 19, 27]. Considering the fulminant differences in treatment and outcome, it is on the one hand risky to rely on radiological diagnostics alone, and on the other hand, the benefits of a modern molecular pathological analysis cannot be used [21, 22, 24].

Especially for lesions in the posterior fossa (brainstem, cerebellum), treatment is often difficult due to a high density of eloquent and vital areas. A total resection of intraaxial lesions in these areas is rarely possible and bears a high risk of complications. The diagnostic success rates of frame-based stereotactic biopsies for posterior fossa lesions are usually high, ranging between about 80 and 95% [4]. Therefore, frame-based stereotactic biopsies are even more important for lesions in the posterior fossa.

Biopsies in the posterior fossa are mainly performed with two different approaches as follows: the transfrontal approach and the suboccipital-transcerebellar approach [3, 4]. The benefit of the suboccipital approach is the short distance between the point of entry and target locus which minimizes the risk of collateral damage to healthy brain tissue and is therefore commonly used, even though it is technically more demanding [16]. Furthermore, publications showed that the complication rates are higher compared to biopsies in supratentorial areas [2, 15, 25].

The published literature focusing on posterior fossa biopsies mostly consists of single-center experiences with a comparatively low number of cases, with case numbers decreasing further when considering only suboccipital-transcerebellar approaches. Considering the high clinical impact and the relatively high risk of complications, additional data with a greater number of cases for analyzing safety and improvement of methods is necessary. In our center, the suboccipital-transcerebellar approach has been widely used for over nearly two decades. In this publication, we want to share our experiences with this approach and focus on diagnostic yield and safety.

## Methods

### Study design

For this retrospective study, all stereotactic biopsies at our hospital from 2007 to March 2023 were screened. All cases with biopsies performed via the suboccipital-transcerebellar approach for posterior fossa lesions were included if the target points were located in the brainstem or cerebellum. Most surgeries were performed by one out of three board-certified neurosurgeons with stereotactic subspecialization.

### Surgical technique

All patients were operated under general anesthesia and with intraoperative stereotactic imaging. Prior to surgery, a single shot of cefazoline (2 g) was infused as prophylactic antibiosis; in the case of penicillin allergy, vancomycin (1.5 g) was used. After orotracheal intubation and initiation of general anesthesia, the patient’s head was fixed in the stereotactic frame (Titanium Frame, Inomed Medizintechnik GmbH, Emmendingen, Germany or a Legacy Carbon Frame, Fischer-Leibinger, Stetten, Germany). The posterior fossa approach requires the frame to be mounted high, in a reverse (negative orientation) fashion with short mounting posts. The frame is mounted parallel to the canthomeatal line with the head fully centered. Fixation pins are placed in the orbital rim and the occipital area. The frame needs to be mounted in a position high enough to allow the blades of the stereotactic localizer to be fitted without colliding with the patient’s shoulders. Three blades are added to the stereotactic frame in the anterior, left, and right positions. Then, the patient is placed in the intraoperative computer tomography (CT) or magnetic resonance imaging (MRI) scanner; in both techniques, images are acquired in the supine position. For intraoperative stereotactic CT scans, the patient is placed with the stereotactic frame attached to the operating table, and the CT scanner is then moved forward on a sliding gantry over the desired area to be scanned. This avoids the need to reposition the patient. For the biopsy, the patient’s head is merely turned laterally exposing the ipsilateral posterior fossa. For intraoperative MR scans, the stereotactic frame is fixed with the patient on an MR-compatible stretcher and is transported to the scanner, which is located in an adjacent room in the operating tract. For the biopsy, the patients are then moved to the operating table, and the frame is fixed to it. The decision, on which modality is used, depends on the age and the comorbidities (e.g., pacemaker) of the patient as well as on the availability of the machines. For intraoperative CT scans, contrast-enhanced 1 mm axial slices were obtained and fused to preselected MRI sequences that visualized the target lesion. For intraoperative MRI scans, a 1-mm contrast-enhanced T1 sequence (T1-VIBE 3D) with isotropic voxels was obtained as the stereotactic image series. In cases of non-contrast-enhancing lesions, a 2-mm sliced FLAIR sequence was added to visualize the target lesion. The localizer blades are removed from the ring, and the patient is placed on the operating table in a supine position. The patient’s head is then rotated to the contralateral side of the planned entry point with the neck slightly inclined to expose the suboccipital area with the ipsilateral shoulder slightly elevated and bolstered. If necessary, the ipsilateral shoulder is gently taped down to increase the working area. The stereotactic frame is then attached to the operating table via a Mayfield-type adapter. The trajectory is then planned with the stereotactic planning software (Inomed Planning System iPS V4.0–7.0, Inomed Medizintechnik GmbH, Emmendingen, Germany). After the stereotactic transformation of the localizer blades, all necessary image sets are automatically fused, and a safe trajectory that avoids conflicts with vessels, CSF spaces, and cerebellar sulci is planned. After shaving the entry point area, the surgical field is thoroughly disinfected and draped in a sterile fashion. The Zamorano-Duchovny (ZD) stereotactic system (Inomed Medizintechnik GmbH, Emmendingen, Germany) is then set to enable the planned trajectory. Settings are checked by two surgeons before mounting the frame in a negative position onto the stereotactic frame. A linear incision of approximately 3 cm is made at the entry point, a small retractor is placed, and bipolar coagulation is performed in the case of any bleeding. To penetrate through the skull, a 12-mm burr hole trephination is made. The dura is coagulated and then opened in a cruciate fashion with a number 15 blade. The underlying cerebellar cortex is avascularized. The biopsy cannula is stereotactically placed with the instrument holder of the ZD stereotactic system set to the depth of the first (most superficial) specimen. The burr hole is then sealed with small portions of gelatin sponge and fibrin glue to minimize loss of cerebrospinal fluid and therefore potential brain shift. Specimens are then taken in a consecutive fashion using stereotactic biopsy forceps (Inomed Medizintechnik GmbH, Emmendingen, Germany) that are advanced in 1-mm steps until the last desired specimen is taken at the target point level. Specimens are then transferred individually into formalin and sent to the Department of Neuropathology for analysis. After the last specimen has been taken, the biopsy cannula is removed, and a gelatin sponge seal is placed inside the burrhole. Wound closure is then performed in a layered fashion before the wound is disinfected and draped. The stereotactic frame is removed prior to termination of general anesthesia and extubation. After surgery, patients stayed under clinical observation at our wards for at least 3 days. A CT scan after the procedure was not performed routinely. The decision to perform a CT scan after surgery depends on the personal decision of the surgeon or medical staff entrusted with the clinical observation on the wards. Every patient with new neurological deterioration received a CT scan.

### 3D model

The 3D Model for visualization of the trajectories was created using the Inomed Planning Software (Inomed Medizintechnik GmbH, Emmendingen, Germany). Coordinates of target- and entry points for each trajectory were determined according to the anterior commissure—posterior commissure (ac-pc) constant. Then, trajectories were inserted in the T1 MRI data set of a healthy, adult male individual, and checked by two surgeons.

### Clinical data

All clinical data were acquired from the hospital records with a focus on surgical reports, discharge letters, admission letters, lab results, and progress reports checked by two surgeons. Histopathological data according to the recent World Health Organization (WHO) classification available at the time of surgery were acquired from the reports of the Department of Neuropathology, Heidelberg University Hospital, Heidelberg, Germany. Radiological data were acquired after image analysis in the Picture Archiving and Communication System (PACS). For detection of complications, the discharge letters as well as the following outpatient letters were critically analyzed regarding new neurological deficits, hemorrhage in postsurgical CT scans, and occurrence of infections or wound healing disorders. A focal neurological deficit (FND) was defined as any worsening of the neurological state of a patient after the procedure. New deficits were classified as “temporary” if the deficit had already been completely resolved at the day of discharge; deficits still existing at the time of discharge were classified as “permanent”. Cases were classified as “non-diagnostic” if a proper histopathological or molecular–pathological diagnosis could not be achieved. These patients were further analyzed regarding the clinical course.

### Statistics

Nominal and ordinal variables are presented as numbers, frequencies, and median; for continuous variables, the mean, range, and standard deviation are shown. Used tests to compare nominal parameters between two groups included the chi-square test and Fisher’s exact test. All statistic was made with SPSS Statistics (IBM, Version 29.0, 2022).

### Ethics

This study was approved by the Ethics Commission of the Heidelberg Medical Faculty, Heidelberg, Germany, and conducted according to the Declaration of Helsinki.

## Results

### Patients' characteristics

From January 2007 to March 2023, a total of *n* = 79 stereotactic biopsies for lesions of the posterior fossa using a suboccipital-transcerebellar approach were performed at our hospital. Overall, 41 patients were female (51.9%) and 38 male (48.1%). The mean age was 42.5 years (range 1–87, standard deviation (SD) 23.3), and 15 patients were pediatric (age under 18, 19%). The most common symptoms were ataxia (55.7%), gait instability (43%), vertigo (44.3%), and diplopia (30.4%). Only five patients (5.3%) had no correlating symptoms and were therefore classified as coincidental. A full list of initial symptoms and ASA scores (American Society of Anesthesiologist Score, ranging from 1 (healthy patient without medical comorbidities, estimated perioperative mortality < 1%) to 5 (moribund patient with a perioperative mortality of 50%) can be found in Table 1.

**Table 1:** Gender, age, symptoms, ASA-scores and radiological parameters of the 79 lesions in our cohort

Parameter	*N*	%
**Gender**	79	100
Male	38	48.1
Female	41	51.9
**Age**	79	100
Adults (> 18)	64	81
Pediatric (< 18)	15	19
Mean age 42.5 (± 23.3)		
**Symptoms**	79	100
Headache	17	21.5
Double vision	24	30.4
Vertigo	35	44.3
Ataxia	44	55.7
Gait instability	34	43
Limb paresis	17	21.5
Cranial nerve paresis	19	24.1
Sensoric deficits	21	26.6
Speech disorder	14	17.7
Hearing disorder	3	3.8
Swallowing disorder	9	11.4
Coincidental finding	5	6.3
**ASA-score**	79	100
1	30	38
2	24	30.4
3	16	20.3
4	9	11.4
**Localization**	79	100
Unifocal	65	82.3
Multifocal	14	17.7
Brainstem involvement	59	74.7
Mesencephalon	11	18.6
Pons	54	91.5
Medulla	20	33.9
Cerebellum involvement	60	75.9
Hemisphere	35	58.3
Peduncle	49	81.7
Vermis	7	11.7
**T1**	78	98.7
Hypointense	54	69.2
Hyperintense	9	11.5
Isointense	15	19.2
**T2**	76	96.2
Homogeneous	47	61.8
Heterogeneous	29	38.2
**Contrast affinity**	77	97.5
Yes	55	71.4
No	22	28.6
**Flair**	76	96.2
Positive	74	97.4
Negative	2	2.6
**Hemorrhage**	77	97.5
Yes	7	9.1
No	70	90.9
**Necrosis**	75	94.9
Yes	16	21.3
No	59	78.7
**Cysts**	77	97.5
Yes	6	7.8
No	71	92.2
**Mass effect**	77	97.5
Yes	47	61
No	30	39
**Hydrocephalus**	77	97.5
Yes	13	16.9
No	64	83.1
**CT**	49	62
Hyperdense	11	22.4
Hypodense	23	46.9
Isodense	15	30.6

### Localization and radiological appearance

A total of 14 cases (17.7%) showed a multifocal appearance with at least one lesion in addition to a posterior fossa lesion, whereas 65 cases (82.3%) appeared unifocal. Brainstem involvement was present in 59 cases (74%) mainly in the pons (54 cases, 91.5%). Cerebellar involvement appeared in 60 cases (75.9%) mainly in the cerebellar pedunculi (49 cases, 81.7%). All localization and radiological data can be found in Table 2.

**Table 2:** Data regarding surgical procedure and outcome. Shown are the number of specimens, the anatomical projections of the target points, CT scan within 7 days after surgery, complications, type of complication, 30-day mortality and 1-year survival after surgery

Parameter	*N*	%
**Modality**	79	100
CT	17	21.5
MRI	62	78.5
**Specimen**	79	100
Minimum	6	
Maximum	31	
Mean specimen 15.7 (± 4.9)		
**Target-point localization**	79	100
Brainstem	36	45.6
Cerebellum	43	54.4
Pons	30	37.9
Mesencephalon	1	1.3
Medulla	5	6.3
Cerebellum peduncle	25	31.6
Cerebellum hemisphere	18	22.8
**CT within 7 days after surgery**	79	100
Yes	25	31.6
No	54	68.4
**Complications**	79	100
Yes	7	8.9
No	72	91.1
**Complication type**	79	100
FND	7	8.9
Transient FND	4	5.1
Permanent FND	3	3.8
Hemorrhage	5	6.3
**30-day mortality**	79	100
Yes	6	7.6
No	73	92.4
**1-year survival**	60	75.9
Yes	42	70
No	18	30

### Trajectories and target points

**Fig. 1:**
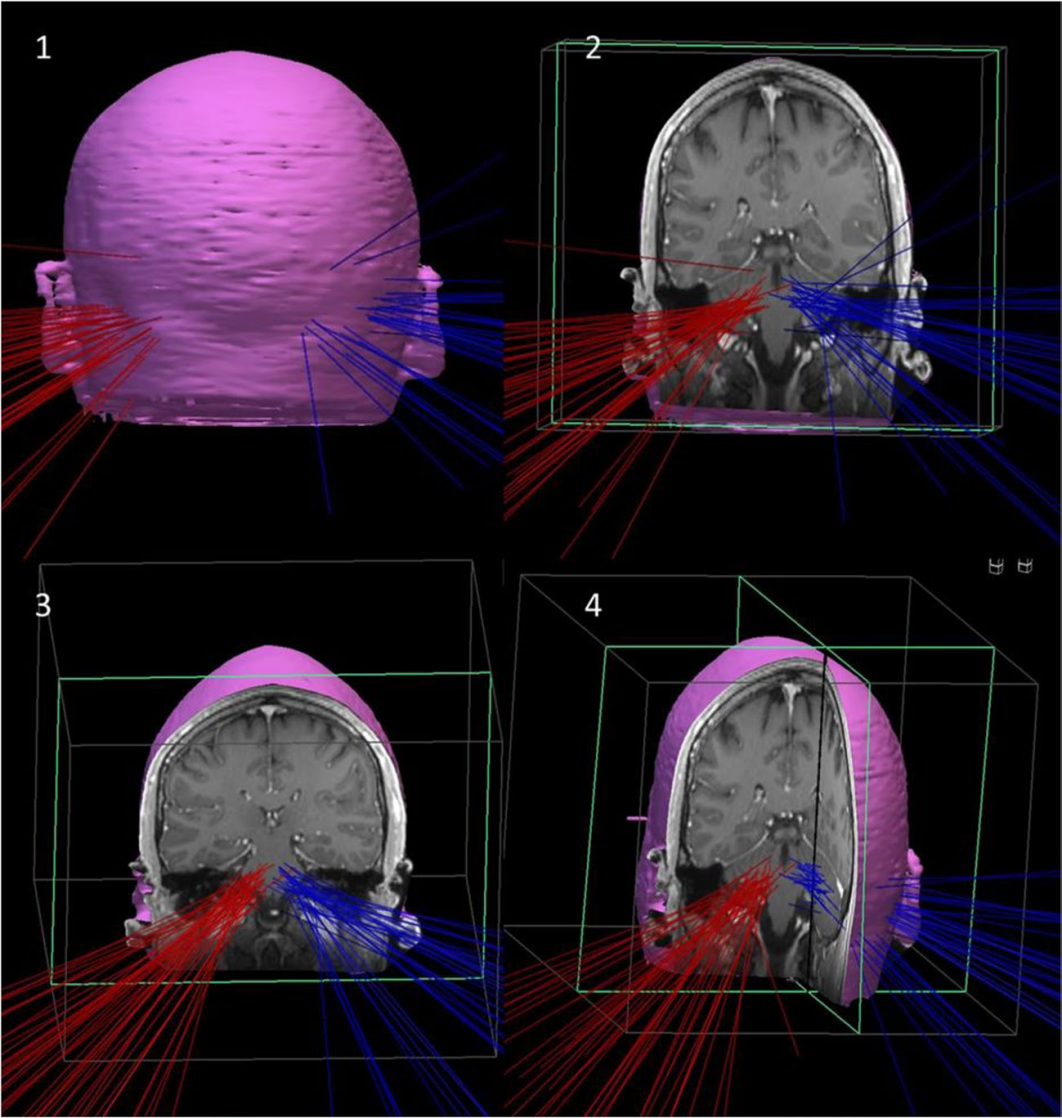
3D model of the used trajectories to reach the lesions in the brainstem and cerebellum via the suboccipital-transcerebellar approach

The course of the trajectories is projected in a 3D model in Fig. 1. Most entry points were located laterally in the suboccipital area. Target points were located in the cerebellum in 43 cases (54.4%) and in the brainstem in 36 cases (45.6%), respectively. Further analyses of the target points showed that the brainstem targets were located in the pons in 30 cases (37.9%), in the mesencephalon in one case (1.3%), and in the Medulla in five cases (6.3%). Cerebellar target points were located in the cerebellar hemispheres in 18 cases (22.8%) and in 25 cases (31.6%) in the cerebellar pedunculi, respectively.

### Diagnostic success rate

In 69 patients, a complete diagnosis could be made enabling a diagnostic yield of 87.3%. Ten cases were lacking a proper diagnosis (12.7%). During the follow-up of these cases, two patients underwent an additional stereotactic biopsy in which a tumor was diagnosed, three patients showed no radiological or clinical progress during follow-up with no need for further intervention, one patient showed progress of glioma at a supratentorial location, two patients are still being followed-up so that a reliable conclusion about the clinical course cannot be made yet, and two patients showed an inconspicuous follow-up, and the goal of the biopsy was solely to rule out a tumor, which could be achieved.

The most common diagnoses were brain tumor (48 cases, 60.8%), lymphoma (ten cases, 12.7%), and inflammatory disease (seven cases, 8.9%). Differentiating the group of brain tumors, we found glioblastoma multiforme WHO 4° (GBM) in 13 cases (16.5%), astrocytoma WHO 2° and WHO 3° in seven cases (8.9%) each, midline glioma in ten cases (12.7%), pilocytic astrocytoma in four cases (5.1%), medulloblastoma in three cases (3.8%), and glioma without the possibility of clear WHO classification in four cases (5.1%). Other diagnoses were M. Alexander, progressive multifocal leukoencephalopathy (PML), and sarcoma, in one case each (1.3%).

When comparing the suspected diagnoses prior to biopsy with the final diagnoses after histopathological analysis, we found a congruence between suspected and true entities in the final diagnosis in only 69.9% of the cases. It is worth mentioning that we only considered the entity of the suspected diagnoses and not the subtype within the entity group.

We found a slight difference between the success rates of biopsies in brainstem lesions (31 of 36, 86.1%) compared to biopsies in cerebellar lesions (38 of 43, 88.4%), but this gap did not appear to be statistically significant (*p* = 0.76).

**Fig. 2:**
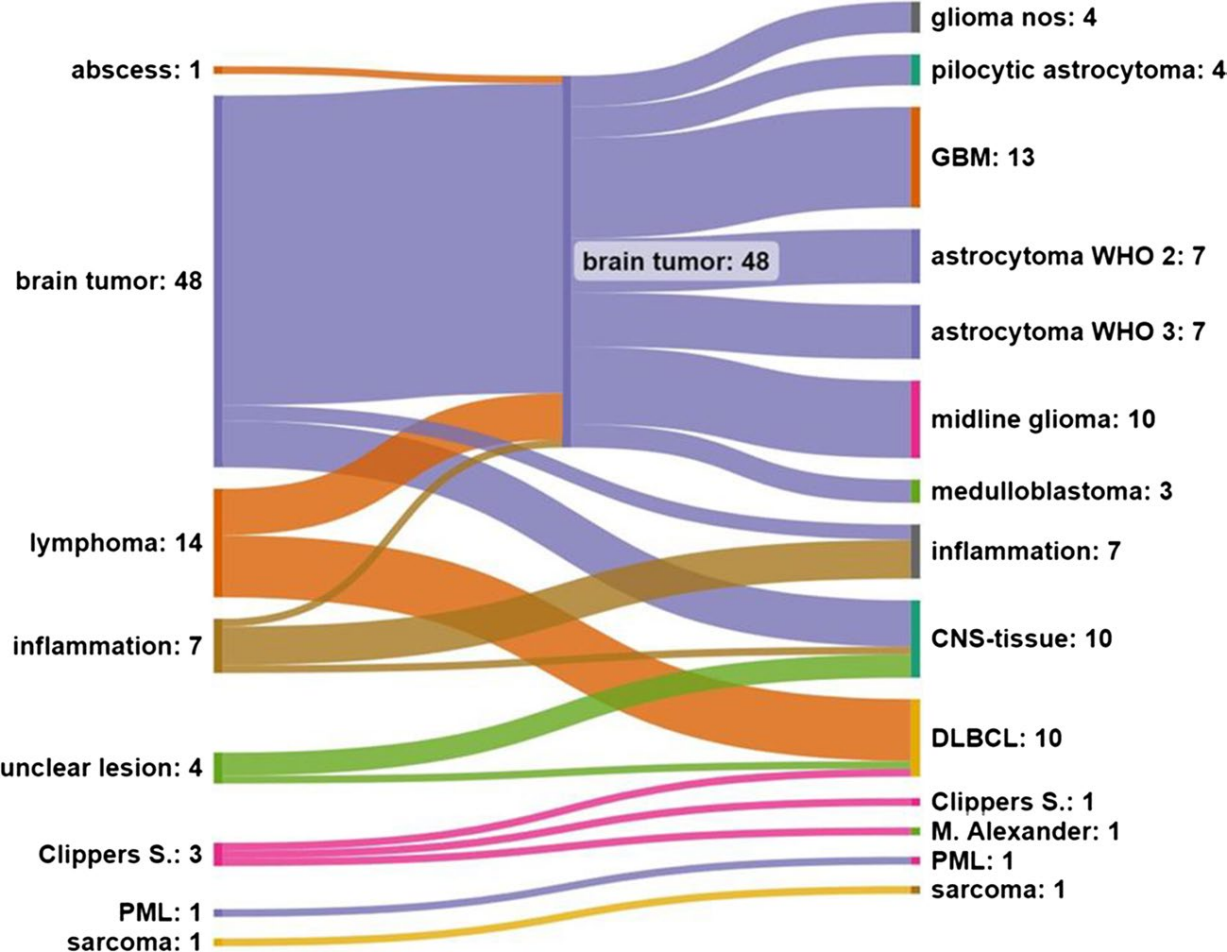
Flowchart of the suspected diagnosis prior to surgery and the end diagnosis after histopathological examination. For the entity “brain tumor”, we also showed the subtype of the tumors. Note that the diagnosis “CNS-tissue” was considered non-diagnostic but is a heterogeneous group of CNS tissue with unspecific findings

Further information as well as a flowchart are available in Fig. 2.

### Complications

Overall, we found surgery-related complications in seven cases, meaning a total complication rate of 8.9%. All patients affected by complications showed new neurological deficits. In four cases (5.1%), these deficits were temporary and had fully regressed at discharge. In three cases (3.8%), the deficits were permanent. In five cases (6.3%) with complications, a post-surgical hemorrhage was detected in CT scans. New neurological deficits consisted of a reduction of vigilance (three cases), aggravation of limb paresis (two cases), and limb hypesthesia and paresis of the seventh cranial nerve (one case each).

While contemplating the target points of cases with complications, we found a complication rate of 13.9% (five cases) for the 36 brainstem biopsies and 4.7% (two cases) for the 43 cerebellar biopsies, respectively. Even though the complication rate for brainstem biopsies appeared higher, we found these disparities not to be significant (*p* = 0.15).

In our cohort, no infections and also no wound-related complications (e.g., wound healing disorders, cerebrospinal fluid leaks) were found. In our cohort, a CT scan within 7 days after surgery was performed in 25 cases (31.7%). In these 25 scans, we found a postsurgical hemorrhage in only five cases (20%), demonstrating that 80% of the CT scans showed normal postsurgical findings without further consequences.

### MRI vs. CT

In our cohort, 62 biopsies (78.5%) were performed using intraoperative MRI, while in 17 cases (21.5%), intraoperative CT was used. We found no significant differences between these two groups in terms of complications (8.1% MRI vs. 11.8% CT, *p* = 0.63) and diagnostic yield (87.1% MRI vs. 88.2% CT, *p* = 0.9).

### Outcome

To assess the outcome after frame-based stereotactic biopsies in the posterior fossa, we analyzed the 30-day mortality after surgery and the 1-year survival rate. In our cohort, six patients (7.6%) died within 30 days after surgery. Those patients showed a median preoperative American Society of Anesthesiologists (ASA)-score of 3.5, compared to 2.0 for patients without death within 30 days, demonstrating a statistically significant difference (*p* < 0.02). Further analyses regarding the cause of the deaths showed an association with the biopsy in 3 cases, where a hemorrhage in the area of the biopsy was found leading to hydrocephalus and herniation. Two patients died due to the progress of the underlying tumor disease, one patient died due to a menigeosis gliomatosa. Data regarding 1-year survival was available for 60 patients. We found a 1-year survival rate of 70% (42 patients). The 18 patients (30%) with deaths within 1 year after biopsy were diagnosed with brain tumors (1-year mortality rate 40.5%) and lymphoma (1-year mortality rate 60%). Patients with other diagnoses survived 1 year in all cases.

## Discussion

### Frame design

**Fig. 3:**
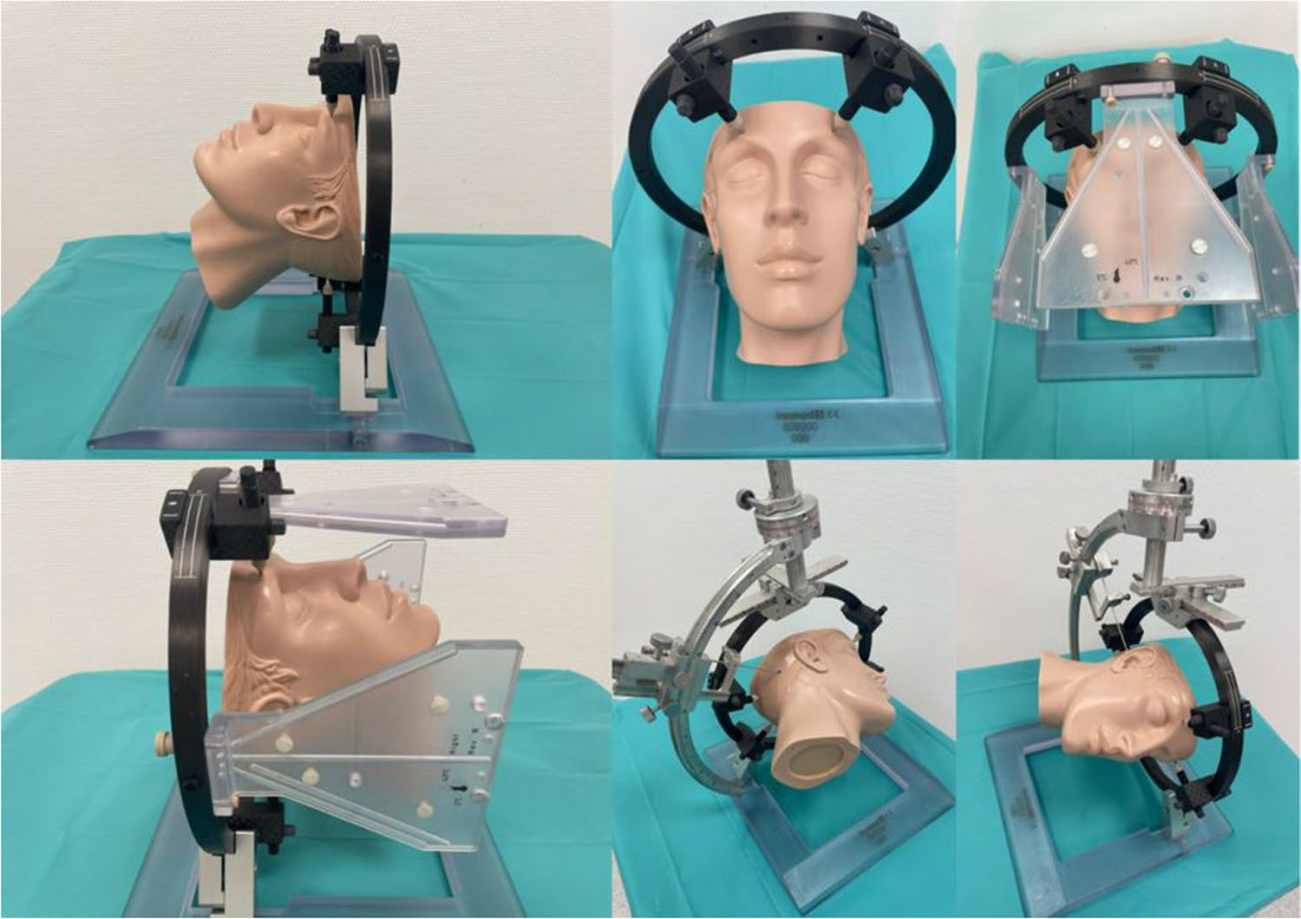
Phantom of the surgical procedure with the stereotactic frame with pins, stereotactic arch, biopsy needle in situ, and localizer blades as well as a demonstration of the patient’s head positioning according to our oblique position for the suboccipital-transcerebellar approach as described detailed in material and methods

**Fig. 4:**
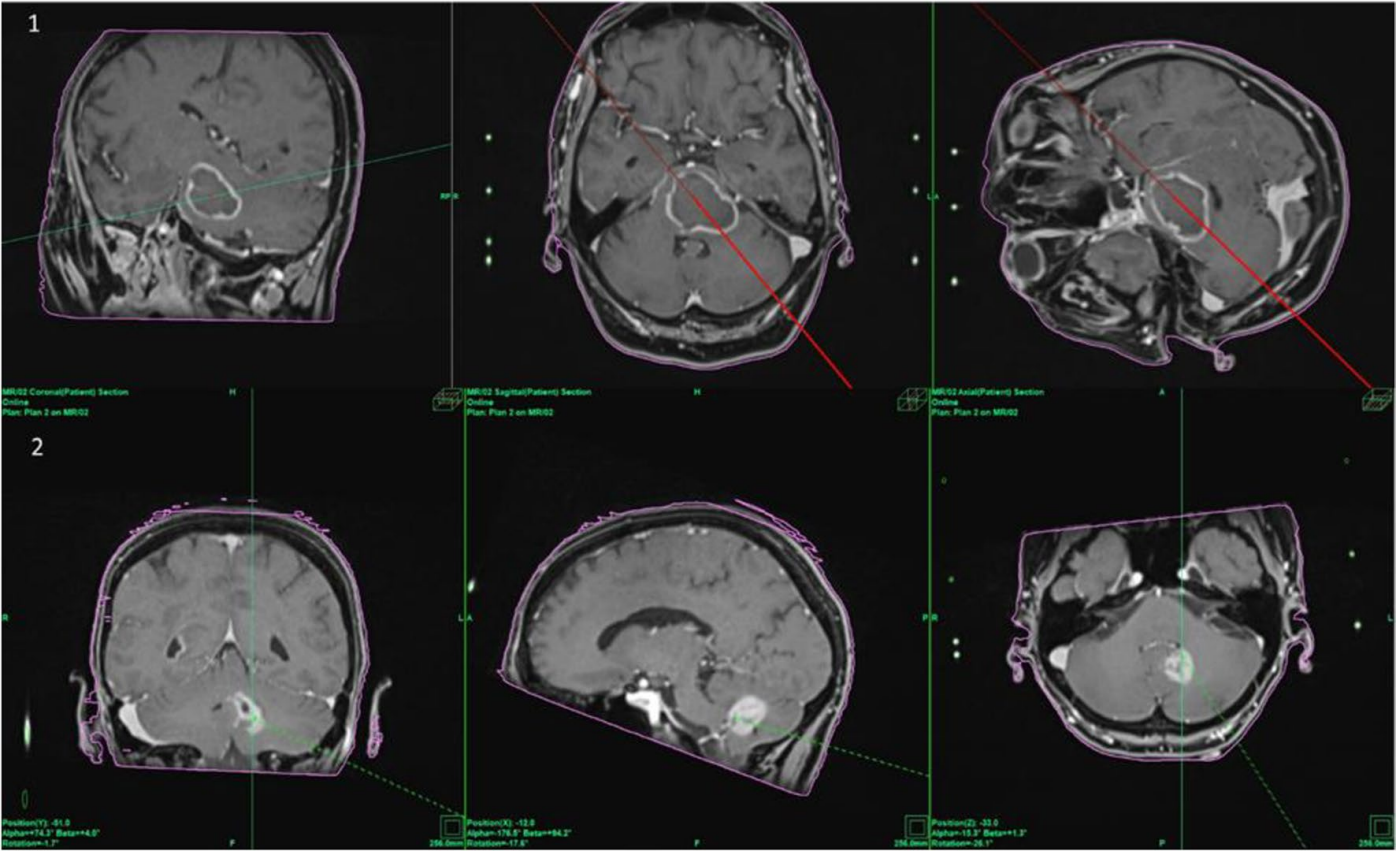
Two illustrative cases of typical lesions a frame-based stereotactic biopsy was performed using the suboccipital-transcerebellar approach. **1** A lesion with circular contrast affinity in the pons is shown. The radiological diagnosis prior to surgery was abscess, but after stereotactic biopsy and tissue analysis, the histological and molecular pathological final diagnosis reported a GBM. **2** A lesion in the deep cerebellar hemisphere with strong contrast affinity is shown. Prior to surgery, the estimated diagnosis was lymphoma, after biopsy and tissue analysis, the final diagnosis revealed a GBM

The ability of the inomed frame to be mounted in an upside-down position means that the mounting posts and pins are positioned superior to the posterior fossa which minimizes the risk of trajectories colliding with parts of the frame. Contrary to other frame systems, the patient can be operated in a supine position with the head turned (Fig. 3), compared to a prone or semi-sitting position which are more time-consuming approaches that require special attention regarding airway management and avoidance of air embolism [4, 9, 16]. Therefore, the Zamorano-Duchovny frame offers a relatively easy patient positioning compared to other frame systems when mounted in an upside-down manner (see also the illustrative cases presented in Fig. 4).

### Diagnostic yield

The diagnostic yield in our cohort was 87.3%. While taking a closer look at the cases lacking proper diagnosis, we found that in two cases, the aim of the biopsy was solely to rule out the possibility of a brain tumor, which was successfully done with the histopathological diagnosis of the central nervous system (CNS) tissue without signs of malignancy. Considering this as a diagnostic success, our diagnostic yield increases to 89.9%. Compared to other published studies, our diagnostic yield ranges in a similar area. Even though there are some studies, mostly with small case numbers, claiming a diagnostic yield of 100%, most studies with a higher case number reported a diagnostic yield ranging between 85 and 95% [4, 5, 10].

While comparing the suspected diagnoses prior to surgery with the histopathological final diagnoses, we found a high incongruence with a match in only approximately 70% of the cases. In this study, we only compared the entities and not even the subtype of the entity. When considering the exact subtype of the hypothesized diagnosis, only a few cases were estimated correctly. This means that there is still a large gap between radiological and histopathological diagnoses, and the need for stereotactic biopsies is still evident. Our findings are in accordance with other published literature, such as Rachinger et al. (2009) and Sanai et al. (2008) [24, 26].

### Safety

Our total complication rate was 8.9% (seven out of 79 cases). Overall, 5.1% showed transient deficits, and 3.8% permanent deficits, respectively. Compared to other published literature, our complication rate is within the typical range [8, 10, 15, 16, 23]. Undoubtedly, the complication rate for brainstem/cerebellar biopsies is higher than at supratentorial target points. We found that the complication rates in brainstem biopsies were higher compared to cerebellar biopsies (13.9% vs. 4.7%) despite not being statistically significant. This might be attributed to the fact that the density of eloquent areas in the brainstem is higher than in the cerebellum.

In our cohort, we found fatal clinical courses, defined as the death of a patient within 30 days after surgery, in six cases (7.6%). It is worth mentioning that the surgery is not the only parameter affecting the 30-day mortality. For example, we found a significantly higher ASA score in patients who died within 30 days compared to those who survived longer than 30 days after surgery (ASA 3.5 vs. ASA 2, *p* < 0.02). So, we hypothesize that the 30-day mortality is more likely affected by other medical conditions and the illness itself. To identify the factors affecting early mortality after stereotactic biopsies, larger studies with a greater number of patients and deeper knowledge of medical conditions are necessary. In our study, only three cases showed a direct link between surgery and complications leading to death. The mortality rate in other published literature ranges from 0 up to 6%. Notably, most studies only considered cases with clear surgery-related deaths. In our study, we provide data regarding early mortality regardless if related to surgery or not.

The 1-year mortality rate in our cohort was 30% and was strongly affected by the histopathological diagnosis. All patients, who died within 1 year after surgery, had diagnoses of either malignant brain tumor or lymphoma. Patients with other diagnoses (e.g., inflammatory disease) all survived 1 year after surgery. Other published literature is in accordance with our findings. For example, Rachinger et al. (2009) reported a similar dependence on histopathological diagnosis and 1-year mortality rate [24]. We think that the 1-year mortality rate is not useful to draw conclusions regarding the safety of the surgery itself, but instead to the clinical course of the underlying disease.

### Intraoperative imaging technique

While comparing the probability of complications and diagnostic yield between the cases using the intraoperative MRI and the intraoperative CT imaging technique, we found no significant differences. Consequently, we hypothesize that the use of intraoperative MRI is not inferior to CT scans fused with preoperative MRI sequences in terms of complications and diagnostic success, in good accordance with other published literature [1]. Therefore, our study underlines the findings of Neumann et al. (2018) where a non-inferiority of MRI-based biopsies compared to CT-based biopsies was initially described with a large number of cases and recently published meta-analyzes showed more evidence regarding the equality of MRI and CT [6, 18]. Our study specified these findings in a sub-cohort and proved intraoperative MRI to be safe to use also for posterior fossa biopsies via the suboccipital approach.

### Postoperative CT scan and complication detection

It is still difficult to differentiate between normal postsurgical changes early after surgery and a pathological finding concerning a postsurgical hemorrhage. While considering our seven patients with new neurological deficits after surgery and the detection of a postsurgical hemorrhage in the following CT scans in five of these patients (meaning the detection of a pathological hemorrhage in 71.4%), we argue that a CT scan should only be performed if the patients are showing neurological deficits post-surgery. Other published literature comes to the conclusion to perform a routine CT scan after 2 h, given the fact that most complications occur within 6 h after surgery [25]. This might be an effective method, especially for centers where the post-surgical observation period is limited. At our center, we observe patients after biopsies routinely for 2–4 days as an in-patient to detect any change in their neurologic state. The best method of postsurgical care for patients after biopsies remains to be elucidated; however, in our cohort, none of the 20 scans performed in patients without new neurological deterioration after surgery showed signs of hemorrhage.

### Future perspectives

There are many more unanswered questions regarding stereotactic biopsies in the posterior fossa as well as future research potential. First of all, due to improvements in radiological methods, the selection of cases that will benefit from a detailed histopathological diagnosis must be continuously investigated to avoid unnecessary complications without the chance of pertinent surplus value to the clinical course. Second, the procedure itself needs to improve regarding safety and efficacy. This could be done by optimizing presurgical planning with improvement in the planning of safe trajectories as well as gathering more evidence on when to use which approach, depending on the localization of the lesion [3, 10]. Therefore, further studies with a higher number of cases are necessary. In modern times, the rise of artificial intelligence (AI) and robot-assisted biopsies are still poorly investigated but could be valuable tools to optimize safety [14, 20].

## Conclusions

In this study, we presented our experiences with frame-based stereotactic biopsies in the posterior fossa (brainstem, cerebellum) via the suboccipital-transcerebellar approach. We found this approach to be of acceptable safety and highly reliable for gathering tissue from posterior fossa lesions for histopathological diagnosis and molecular analysis.

## Data Availability

The dataset generated and analyzed during this study are available from the corresponding author upon reasonable request.
